# Differential SLC6A4 methylation: a predictive epigenetic marker of adiposity from birth to adulthood

**DOI:** 10.1038/s41366-018-0254-3

**Published:** 2019-01-08

**Authors:** Karen A. Lillycrop, Emma S. Garratt, Philip Titcombe, Phillip E. Melton, Robert J. S. Murray, Sheila J. Barton, Rebecca Clarke-Harris, Paula M. Costello, Joanna D. Holbrook, James C. Hopkins, Caroline E. Childs, Carolina Paras-Chavez, Philip C. Calder, Trevor A. Mori, Lawrie Beilin, Graham C. Burdge, Peter D. Gluckman, Hazel M. Inskip, Nicholas C. Harvey, Mark A. Hanson, Rae-Chi Huang, Cyrus Cooper, EpiGen Consortium, Keith M. Godfrey

**Affiliations:** 1 Centre for Biological Sciences, Faculty of Natural and Environmental Sciences, University of Southampton, Southampton, UK; 2 NIHR Southampton Biomedical Research Centre, University of Southampton and University Hospital Southampton NHS Foundation Trust, Southampton, UK; 3 Academic Unit of Human Development and Health, Faculty of Medicine, University of Southampton, Southampton, UK; 4 MRC Lifecourse Epidemiology Unit, University of Southampton, Southampton, UK; 5 Centre for Genetics of Health and Disease, University of Western Australia, Perth, Australia; 6 Faculty of Health Science, Curtin University, Perth, WA Australia; 7 School of Medicine, University of Western Australia, Perth, WA Australia; 8 Liggins Institute, University of Auckland, Auckland, New Zealand; 9 Telethon Kids Institute, University of Western Australia, Perth, WA Australia; 10 NIHR Biomedical Research Centre, University of Oxford, Oxford, UK

**Keywords:** Epidemiology, Translational research, Development

## Abstract

**Background:**

The early life environment may influence susceptibility to obesity and metabolic disease in later life through epigenetic processes. SLC6A4 is an important mediator of serotonin bioavailability, and has a key role in energy balance. We tested the hypothesis that methylation of the *SLC6A4* gene predicts adiposity across the life course.

**Methods:**

DNA methylation at 5 CpGs within the *SLC6A4* gene identified from a previous methyl binding domain array was measured by pyrosequencing. We measured DNA methylation in umbilical cord (UC) from children in the Southampton Women’s Survey cohort (*n* = 680), in peripheral blood from adolescents in the Western Australian Pregnancy Cohort Study (*n* = 812), and in adipose tissue from lean and obese adults from the UK BIOCLAIMS cohort (*n* = 81). Real-time PCR was performed to assess whether there were corresponding alterations in gene expression in the adipose tissue.

**Results:**

Lower UC methylation of CpG5 was associated with higher total fat mass at 4 years (*p* = 0.031), total fat mass at 6–7 years (*p* = 0.0001) and % fat mass at 6–7 years (*p* = 0.004). Lower UC methylation of CpG5 was also associated with higher triceps skinfold thickness at birth (*p* = 0.013), 6 months (*p* = 0.038), 12 months (*p* = 0.062), 2 years (*p* = 0.0003), 3 years (*p* = 0.00004) and 6–7 years (*p* = 0.013). Higher maternal pregnancy weight gain (*p* = 0.046) and lower parity (*p* = 0.029) were both associated with lower SLC6A4 CpG5 methylation. In adolescents, lower methylation of CpG5 in peripheral blood was associated with greater concurrent measures of adiposity including BMI (p ≤ 0.001), waist circumference (*p* = 0.011), subcutaneous fat (p ≤ 0.001) and subscapular, abdominal and suprailiac skinfold thicknesses (*p* = 0.002, *p* = 0.008, *p* = 0.004, respectively). In adipose tissue, methylation of both *SLC6A4* CpG5 (*p* = 0.019) and expression of *SLC6A4* (*p* = 0.008) was lower in obese compared with lean adults.

**Conclusions:**

These data suggest that altered methylation of CpG loci within *SLC6A4* may provide a robust marker of adiposity across the life course.

## Introduction

Obesity is a disorder of energy balance resulting from a combination of genetic, lifestyle and environmental factors, and is a major risk factor for non-communicable diseases including type-2 diabetes and cardiovascular disease. Polymorphisms in several genes have been linked to obesity [1–3], but to date genetic variation only accounts for a modest proportion (<10%) of obesity risk at the population level [4, 5]. Epidemiological and experimental studies suggest that susceptibility to adiposity and metabolic disease in later life is influenced by the early life environment [6]. In animal studies, variation in maternal body composition and diet leads to alterations in metabolism and body composition in the offspring [7], while in humans maternal obesity and excess gestational weight gain independently increase adiposity and risk of obesity in the infant, child and adult [8]. The mechanisms underlying these relationships are poorly understood, but modulation of the epigenome, in particular DNA methylation, is thought to be a key mechanism.

Epigenetic processes, which include DNA methylation, histone modifications and non-coding RNAs, induce heritable changes in gene expression without altering the gene sequence. In animal models, variations in the maternal diet induces epigenetic changes in a range of metabolic control genes, this is accompanied by changes in gene expression and metabolism in the offspring [9–11]. Human studies have also suggested an important role for epigenetic processes in mediating the effects of early life environment. For example, methylation of a CpG within the promoter of *RXRA* in umbilical cord predicted >25% of the variation in percent and absolute fat mass in children aged 9 years, with replication being found in a second independent cohort of 6 year old children [12]. In a separate study, methylation of specific CpG loci in the promoter of *PGC1α* at 5–7 years of age predicted adiposity year-on-year from 8 to 14 years [13]. These findings support the hypothesis that epigenetic marks induced during development make an important contribution to phenotype and suggest that detection of such marks in peripheral tissues may provide predictive markers of later phenotype.

To identify further epigenetic marks at birth associated with later adiposity, we previously carried out a discovery scan of DNA methylation in the promoters of all refseq genes in umbilical cord DNA using a methyl capture array (MBD-array). We aimed to identify methylation differences in umbilical cord DNA at birth associated with percentage fat mass in children aged 6 years. Our analyses identified 93 differentially methylated regions (DMRs) [14], including a DMR within the first intron of the *SLC6A4* gene, which encodes the serotonin transporter. SLC6A4 has a major role in modulating the bioavailability of the neurotransmitter serotonin [15], which is synthesised both peripherally and in the central nervous system (CNS), and has a key role in modulating mood, anxiety, and energy homoeostasis [16, 17]. Polymorphisms within *SLC6A4* have been associated with obesity in children [18] and adults [19], while differential methylation of the promoter region of *SLC6A4* in peripheral blood has been associated with concurrent obesity in adults [20].

Given the central role that *SLC6A4* has in energy homoeostasis, and the previous genetic and epigenetic associations with obesity, this study sought to examine the relationship between *SLC6A4* DMR methylation in cord tissue at birth and adiposity in infancy and childhood in children from the Southampton Women’s Survey (SWS) cohort (*n* = 680). Here, we report an association between the methylation of CpG loci within the *SLC6A4* gene at birth and measures of adiposity from birth through to 6 years of age. To assess whether this effect is sustained through the life course, we additionally measured *SLC6A4* DMR methylation in peripheral blood mononuclear cells (PMBCs) from adolescents aged 17 years in the RAINE study and in subcutaneous adipose tissue in lean and obese adults from the BIOCLAIMS cohort. We found that *SLC6A4* CpG methylation at Hg19 chr17:28561468 was associated with adiposity in adolescents (*n* = 812) and in adipose tissue from obese vs. lean adults (*n* = 81), suggesting that altered methylation of CpG loci within *SLC6A4* may provide a robust marker of adiposity across the life course.

## Materials and methods

### Southampton Women’s Survey (SWS) Cohort

The SWS is a prospective mother-offspring cohort study that has assessed the diet, body composition, physical activity and social circumstances of non-pregnant women aged 20–34 years living in Southampton, UK. Comprehensive details of SWS have been published [21, 22]. Follow-up of the children and sample collection/analysis was carried out under Institutional Review Board approval (Southampton and South West Hampshire Research Ethics Committee) with written informed consent. Offspring adiposity was measured by dual-energy X-ray absorptiometry (DXA) at birth, 4 and 6–7 years of age and triceps skinfold thickness at birth, 6 months, 1, 2, 3 and 6–7 years of age. Table 1 shows cohort characteristics.

**Table 1:** Characteristics of the study participants for the SWS, RAINE and BIOCLAIMS cohorts

SWS characteristic	% or median (5th, 95th percentile) for SWS cohort (*n* = 680)	RAINE characteristic	% or median (5th, 95th percentile) for Raine Study year 16 cohort (*n* = 1121)	BIOCLAIMS characteristic	% or median (5th, 95th percentile) for BIOCLIAMS cohort (*n* = 65)
**Mother**		**Mother**			
**Education**		**Education**			
None	2.10%				
CSE	8.70%	School	46.80%		
O levels	26.40%	Trade certificate or apprenticeship	8.10%		
A levels	31.90%	Professional registration (non-degree)	10.50%		
HND	7.80%	College diploma or degree	17.50%		
Degree	23.00%	University degree	11.90%		
		Other	5.20%		
**Social class**		**Family income at 18 weeks GA**			
Unskilled	1.20%	<$7000	6.70%		
Partly skilled	10.80%	$7000–12,000	8.00%		
Skilled manual	6.80%	$12,000–$24,000	22.40%		
Skilled non-manual	35.10%	$24,000–$36,000	27.70%		
Management and technical	40.10%	>$36,000	35.20%		
Professional	6.00%				
Age at birth, years	31.5 (24.6, 36.5)	Age at birth (years)	29 (19–38)		
Smoking (during pregnancy)	12.90%	Smoking (during pregnancy)	21.30%		
Pre-pregnancy BMI	24.2 (19.6, 34.8)	BMI	21.4 (17.6–31.7)		
Pregnancy weight gain (kg/wk)	0.35 (0.07, 0.67)				
**Child**		**Child**		**Adult**	
				**Lean group**	
Female	50.20%	Female	49.00%	Female	82.70%
Birth order		Birth length, cm	49.0 (44.7–53.0)	**Obese group**	
1st	47.90%	Birth head circumference, cm	34.5 (31.5–37.0)	Female	86.30%
2nd	38.20%	BMI age 16	22.1 (18.0–32.7)		
3rd or higher	13.80%				
Birth weight, kg	3.5 (2.7, 4.4)	Birth weight, kg	3.4 (2.3–4.2)		
Gestational age, weeks	40.1 (37.0, 41.9)				
**Age of child at evaluation**		**Age of child at evaluation**		**Age at evaluation**	
Birth triceps (days)	1.00 (0, 2.00)	Cohort	17.04 (18.31 max–16.01 min)	Obese group	41.7 (23.2–60.5)
6 months triceps (years)	0.52 (0.47, 0.63)			Lean group	27.1 (19.2–62.1)
12 months triceps (years)	1.03 (0.98, 1.14)				
2 years triceps (years)	2.03 (1.97, 2.13)				
3 years triceps (years)	3.04 (2.98, 3.22)				
6 years triceps (years)	6.70 (6.25, 7.16)				
Baby DXA (days)	6.00 (1.00, 14.00)				
4 year DXA (years)	4.11 (4.03, 4.23)				
6 year DXA (years)	6.81 (6.35, 7.41)				
**Offspring anthropometry**		**Offspring anthropometry age 17**		**Anthropometry**	
				**Lean group**	
Total fat at birth, g	510.3 (264, 979)	Skinfolds subscapular, mm	12.1 (6.9–29.4)	Weight, kg	59.0 (47.8–78.0)
4 yrs, kg	4.1 (2.8, 6.7)	Suprailiac, mm	13.8 (5.2–33.8)	Height, m	1.66 (1.57–1.84)
6 yrs, kg	4.9 (3.0, 8.8)	Abdominal, mm	19.9 (7.0–39.3)	BMI, kg/m^2^	31.7 (19.5–39.9)
Percentage fat at birth	14.4 (8.8, 22.5)	Waist circumference, cm	77.0 (65.7–102.8)	Waist, cm	74.6 (68.4–87.6)
4 yrs	28.9 (21.7, 39.1)	Hip, cm	96.2 (85.7–114.6)	Hip, cm	94.4 (81.6–101.5)
6 yrs	25.2 (17.7, 35.8)			Percentage fat	26.2 (12.8–31.5)
Triceps skinfold at birth, mm	4.7 (3.4, 6.3)			Fat mass, kg	15.3 (9.0–20.9)
6 months, mm	11.0 (7.8, 15.0)			Total body water, kg	31.1 (26.9–46.6)
12 months, mm	10.9 (7.5, 15.2)			Lean mass, kg	42.4 (36.7–63.5)
2 yrs, mm	10.0 (6.9, 13.7)			**Obese group**	
3 yrs, mm	9.9 (6.8, 13.9)			Weight, kg	97.8 (80.8–126.3)
6 yrs, mm	9.5 (6.3, 16.8)			Height, m	1.68 (1.5–1.8)
				BMI, kg/m^2^	34.5 (30.2–40.0)
				Waist, cm	106.8 (93.7–128.4)
				Hip, cm	117.2 (103.7–134.3)
				Percentage fat	43.0 (27.7–49.7)
				Fat mass, kg	40.1 (25.6–55.9)
				Total body water, kg	38.8 (33.7–60.4)
				Lean mass, kg	53.0 (46.0–82.5)
**DNA methylation**		**DNA methylation**		**DNA methylation**	
*SLC6A4* CpG1	83.8 (76.7–88.23)				
*SLC6A4* CpG2	84.7 (79.9–88.3)				
*SLC6A4* CpG3	84.5 (75.1–89.4)				
*SLC6A4* CpG4	87.5 (78.1–91.9)				
*SLC6A4* CpG5	79.5 (68.7–86.3)	*SLC6A4* CpG5	53.5 (38.9–64.3)	*SLC6A4* CpG5	60.39 (43.18–71.02)
				*SLC6A4* CpG5 Lean Only	63.47 (44.91–72.46)
				*SLC6A4* CpG5 Obese Only	56.62 (41.75–70.43)

### The West Australian Pregnancy Cohort (RAINE) Study

The RAINE Study enroled pregnant women ≤ 18 weeks gestation (1989–1991) through the antenatal clinic at King Edward Memorial Hospital and nearby private clinics in Perth, Western Australia. Detailed clinical assessments were performed at birth (*n* = 2868) and the children followed up at multiple time points including at 17 years of age, when a blood sample was taken, and waist/hip circumference, skinfold thickness and abdominal (subcutaneous and visceral) adipose thickness measurements made [23]. The Human Ethics Committees of King Edward Memorial Hospital and Princess Margaret Hospital approved all protocols (Table 1). Informed, written consent to participate in the study was obtained from the mother of each child at enrolment and at each subsequent follow-up.

### BIOCLAIMS Cohort

The BIOCLAIMS study was a randomised controlled clinical trial of male and female volunteers aged 18–65 years recruited in Southampton UK 2012–2013 (http://bioclaims.uib.eu). The study was approved by the National Research Ethics Service Committee South Central Berkshire. Written informed consent was obtained from all subjects. The study was registered at www.isrctn.com as ISRCTN96712688. Subcutaneous adipose tissue biopsies were collected from 81 volunteers at baseline (lean *n* = 37, obese *n* = 44). Participants were grouped as lean or obese according to their BMI: Lean participants were determined as those with a BMI 18.5–25 kg/m^2^, whilst obese participants had a BMI 30–40 kg/m^2^ with waist circumference > 94 cm for men and >80 cm for women (Table 1).

### DNA extraction

For SWS offspring, genomic DNA was prepared by a high-salt method from a 5 to 10 cm segment cut from the mid portion of the umbilical cord [14]. Genomic DNA was extracted from peripheral blood (RAINE cohort) using the Puregene DNA isolation kit (Qiagen, Germany) and from adipose tissue (BIOCLAIMS cohort) using the QIAamp DNA mini kit (Qiagen, Germany).

### Pyrosequencing

**Fig. 1:**
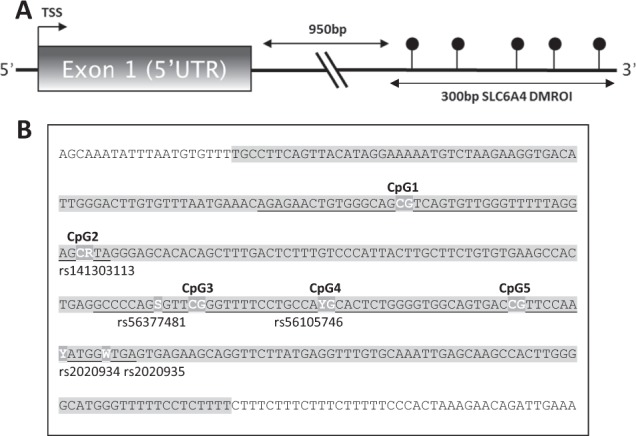
Location of the *SLC6A4* DMR. Location of the *SLC6A4* DMR and known genetic variants is shown in relation to the *SLC6A4* gene. **a** The location of the *SLC6A4* DMR (GRCh37/hg19 Chr17:2856138128561680negative strand) is shown in relation to the first exon of SLC6A4. **b** The location of the CpG’s measured by pyrosequencing and nearby characterised genetic variants is shown. The DMR is shown in grey (reverse strand Chr17:28561381-28561680). The underlined region indicates the region sequenced by Pyrosequencing (either SNP or methylation assay)

Bisulphite conversion and pyrosequencing reactions were carried out [24] using primers listed in Table S1, which were designed across the 5 CpG sites within the *SLC6A4* DMR (TSS + 1450 bp, Hg19 Chr:17(−) 28561505-28561468) identified previously [14] (Fig. 1a). Summary statistics for SLC6A4 methylation shown in Table S2. To identify potential transcription factor binding sites across the CpG sites within the SLC6A4 DMR Matinspector analysis was carried out (omictools.com/matinspector-tool).

### Genotyping analysis

Genotyping PCR reactions (Fig. 1b) on SWS and BIOCIAIMS participants used 50 ng genomic DNA extracted from UC and adipose tissue, respectively (Table S1), and were analysed using PyroMark MD 1.0 software (Biotage). Genotyping of the RAINE participants used 250 ng DNA on the Illumina Human 660-W Quad Array (San Diego, CA) at the Centre for Applied Genomics (Toronto, Canada). Additional imputation using Mach 1.0.18.c [25] for pre-phasing and MiniMac [26] for imputation against reference panel 1000G Phase3 data. A total of 97,718 SNPs were removed at genotyping QC due to a call rate < 0.95%, 919 SNPs were removed due to Hardy–Weinberg equilibrium < 5.7 × 10E−7, and 35,502 SNP with a MAF < 0.05 were removed.

### RT-PCR

Adipose tissue was processed using the RNeasy Lipid Tissue kit (Qiagen, Germany) according to manufacturer’s instructions. A total of 500 ng RNA was incubated with 10 µM random nonamers, 0.5 µM dNTP’s and 200 U M-MLV RT, followed by cDNA amplification using the QuantiTect SLC6A4 primers (Qiagen, Germany).

### Statistical methods

Statistical analysis was carried out using Stata (Statacorp) versions 13.1/14.0/14.2. Distributions were examined prior to analysis, and post analysis diagnostic checks were performed where necessary to ensure assumptions were not violated. All outcome and predictor variables were standardised to a mean of zero and standard deviation (SD) of one, for ease of interpretation. Adiposity was measured as % fat and total fat, and methylation was measured as % methylation, with regression coefficients (*β*) representing the SD change in % fat (or total fat) for each SD change in methylation (Table S2). The skinfold thickness measurements used at each age were calculated using the mean of three skinfold measures, with regression coefficients (*β*) representing the standard deviation change in skinfold thickness for each SD change in methylation. Where appropriate, measures of adiposity and skinfold thickness were transformed to satisfy the assumption of normality. Regression models were built using child’s fat mass, % fat mass or skinfold thickness as outcomes, and CpG methylation as the predictor. All results are presented as regression coefficients (*β*) with their associated *p*-values and 95% confidence intervals. Statistical comparisons of gene expression, or CpG methylation with the lean and obese groups from the BIOCLAIMS cohort was by logistic regression. Analyses were adjusted for sex, and for age where appropriate.

Two of the *SLC6A4* SNPs (rs2020934 and rs2020935) were analysed to assess their impact on results. Where possible, both additive and co-dominant models were considered. Mann–Whitney/Kruskal–Wallis tests were performed on CpG 1-5 for rs2020934 and rs2020935, respectively. DXA total fat (at birth, 4 and 6 years of age) was the outcome used as the primary measure of child’s fat mass across all ages in the following regressions; our regression models were adjusted (i) for SNPs individually and (ii) for both rs2020934 and rs2020935 together.

## Results

### SWS cohort characteristics

Genomic DNA was extracted from the umbilical cord of SWS infants (*n* = 680) who had measurements of adiposity using DXA or triceps skinfold thickness during infancy and childhood. The infants had a median birth weight of 3.48 kg and gestational age of 40.1 weeks; 50% were female. Median maternal age at birth was 31.5 years, pre-pregnancy median body mass index (BMI) 24.3 kg/m^2^ and pregnancy weight gain 0.35 kg/week; 48% were in their first pregnancy and 12% smoked in late pregnancy. Table 1 shows additional characteristics and median (5th–95th percentile) cord tissue *SLC6A4* methylation values for CpGs 1-5.

### Umbilical cord *SLC6A4* CpG methylation and percent and total fat mass in infancy/childhood

**Fig. 2:**
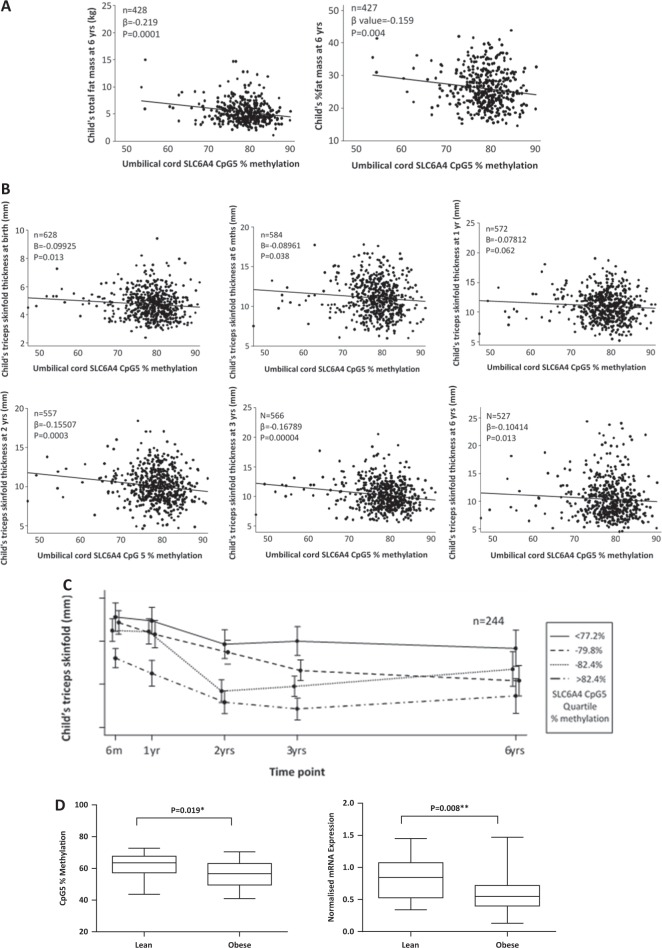
Lower methylation of *SLC6A4* CpG5 is associated with increased fat mass in the SWS and BIOCLAIMS cohorts. **a** Association between the methylation of SLC6A4 CpG5 total and % fat mass at 6 years of age. Means are plotted for each individual. **b** Associations between the methylation of SLC6A4 CpG5 at birth and triceps skinfold thickness at birth, 6 months, 1, 2, 3 and 6 years of age. Means are plotted for each individual. **c** Trajectory of triceps skinfold thickness from 6 months to 6 years with respect to quarters of SLC6A4 CpG5 methylation. Values are age and sex adjusted (*n* = 244). Means ± SEM. **d** Adipose tissue SLC6A4 CpG5 methylation (*n* = 51) and expression (*n* = 61) is shown for lean and obese individuals from the BIOCLAIMS cohort. Whiskers show min to max. *p = 0.01–0.05, **p ≤ 0.01

Examining the relationship between cord tissue *SLC6A4* methylation at birth and infant/child adiposity, lower CpG1 (Hg19:28561601) and CpG2 (Hg19:28561578) methylation were associated with lower % fat mass age 6–7 years (CpG1, *β* = 0.118 (95% CI = 0.015, 0.221), *p* = 0.025; CpG2, *β* = 0.091 (0.0001, 0.183), *p* = 0.05, respectively) (Table 2), but were not associated with % fat mass at birth or 4 years or with total fat mass at birth, 4 or 6–7 years. In contrast, lower CpG5 (Hg19:28561468) methylation was associated with higher % fat mass at 6–7 years (*β* = −0.159 (−0.267, −0.052), *p* = 0.004). Lower CpG5 methylation was also associated with higher total fat mass at birth (*β* = −0.102 (−0.213, 0.009), *p* = 0.072), 4 years (*β* = −0.108 (−0.206, −0.010), *p* = 0.031) and 6–7 years (*β* = −0.219 (−0.325, −0.113), *p* = 0.0001) (Table 2, Fig. 2a). There were no associations between the methylation of CpG3 (Hg19:28561505) or CpG4 (Hg19:28561490) and % or total fat mass at any age.

**Table 2:** Associations between umbilical cord *SLC6A4*CpG methylation levels and child’s fat mass in the SWS cohort

Phenotype	CpG1 (standardised)	CpG2 (standardised)	CpG3 (standardised)	CpG4 (standardised)	CpG5 (standardised)
DXA: total fat at birth (standardised): adjusted for sex, age and gestational age					
*n*	301	294	302	299	299
*β*	0.063	−0.014	−0.099	−0.132	−0.102
*p*-value	0.341	0.837	0.086	0.136	0.072
95% CI	(−0.067, 0.194)	(−0.146, 0.118)	(−0.212, 0.014)	(−0.306, 0.042)	(−0.213, 0.009)
DXA: %fat at birth (standardised): adjusted for sex, age and gestational age					
*n*	301	294	302	299	299
*β*	0.079	−0.022	−0.084	−0.096	−0.075
*p*-value	0.232	0.74	0.141	0.271	0.177
95% CI	(−0.051, 0.208)	(−0.153, 0.109)	(−0.195, 0.028)	(−0.268, 0.075)	(−0.185, 0.034)
DXA: total fat at 4 years (standardised): adjusted for sex					
*n*	414	400	413	409	405
*β*	−0.037	0.028	0.007	0.024	−0.108
*p*-value	0.498	0.558	0.89	0.75	0.031*
95% CI	(−0.145, 0.071)	(−0.065, 0.121)	(−0.089, 0.102)	(−0.123, 0.172)	(−0.206, −0.010)
DXA: %fat at 4 years (standardised): adjusted for sex					
*n*	414	400	413	409	405
*β*	0.01	0.011	−0.02	0.046	−0.081
*p*-value	0.854	0.814	0.682	0.536	0.104
95% CI	(−0.098, 0.118)	(−0.082, 0.104)	(−0.115, 0.075)	(−0.101, 0.194)	(−0.179, 0.017)
DXA: total fat at 6 years (standardised): adjusted for sex and age					
*n*	435	427	432	430	428
*β*	0.039	0.073	−0.028	−0.058	−0.219
*p*-value	0.451	0.112	0.575	0.438	0.0001**
95% CI	(−0.063, 0.141)	(−0.017, 0.164)	(−0.126, 0.070)	(−0.204, 0.089)	(−0.325, −0.113)
DXA: %fat at 6 years (standardised): adjusted for sex and age					
*n*	434	426	431	429	427
*β*	0.118	0.091	−0.024	−0.037	−0.159
*p*-value	0.025*	0.049*	0.628	0.621	0.004**
95% CI	(0.015, 0.221)	(0.0001, 0.183)	(−0.123, 0.074)	(−0.183, 0.110)	(−0.267, −0.052)

Associations between %/total fat mass at birth, 4 and 6 years, adjusted for *SLC6A4* batch effect. 95% confidence limits (Cl) are shown

*p ≤ 0.01–0.05

**p ≤ 0.01

Bold values indicate a *p* value lower than 0.05

Multivariate analysis of DXA % fat and total fat mass at birth, 4 and 6 years of age adjusted for sex and age showed that methylation of CpG5 was associated with total fat mass at birth (*β* = −0.1261 (−0.250, −0.002), *p* = 0.046), 4 (*β* = −0.1104 (−0.215, −0.006), *p* = 0.039) and 6 years (*β* = −0.2039 (−0.314, −0.094), *p* = 0.0003) of age and with % fat mass (*β* = −0.1447 (−0.257, −0.033), *p* = 0.011) at 6 years; there was no association however between CpG2 methylation and % or total fat mass at any time point (Table S3). The effect size was such that for each standard deviation increase in methylation of *SLC6A4* CpG5, there was an average decrease of 0.219 standard deviations in total fat mass at 6–7 years of age, accounting for sex, age and batch effect. Additional adjustments for smoking in pregnancy, parity, IOM pregnancy weight gain and mother’s pre-pregnancy BMI did not substantially alter the association between CpG5 methylation and total fat mass at 4 and 6 years but did weaken the association with total fat at birth (adjusted *β* = 0.0099 (−0.124,0.144), *p* = 0.884) and % fat mass at age 6 years (adjusted *β* = −0.0986 (−0.214,0.017), *p* = 0.0946).

### Umbilical cord *SLC6A4* methylation and triceps skinfold thickness from birth to age 6–7 years

To determine whether cord *SLC6A4* methylation was associated with additional measures of adiposity in childhood, we investigated the relationship between *SLC6A4* methylation and triceps skinfold thickness in the SWS children from birth through to 6–7 years. Lower methylation of CpG5 was associated with higher triceps skinfold thickness at birth (*β* = −0.0993 (−0.177, −0.021), *p* = 0.013,), 6 months (*β* = −0.090 (−0.174, −0.005), *p* = 0.038,), 12 months (*β* = −0.0781 (−0.160, 0.004), *p* = 0.062), 2 years (*β* = −0.155 (−0.238, −0.072), *p* = 0.0003), 3 years (*β* = −0.168, (−0.248, −0.088), *p* = 0.00004) and 6 years (*β* = −0.104 (−0.186, −0.022), *p* = 0.013) (Table S4, Fig. 2b). Tracking the association of *SLC6A4* CpG5 methylation with skinfold thickness from birth to age 6–7 years, infants with the lowest quarter of *SLC6A4* methylation at birth had, on average, the highest triceps skinfold thickness at each of the ages from birth to 6–7 years, while individuals with the highest quarter of *SLC6A4* methylation had, on average, the lowest skinfold thickness from birth to 6–7 years (Fig. 2c). Lower CpG4 methylation was associated with higher triceps skinfold thickness at birth (*β* = −0.137 (−0.254, −0.019), *p* = 0.023) but there were no associations at later ages. There were no associations between the methylation of CpGs 1, 2 or 3 and triceps skinfold thickness at any age.

### Effects of genetic variation within the *SLC6A4* DMR on methylation of *SLC6A4* DMCpGs in the SWS cohort

Genotyping analysis in all participants excluded the presence of genetic variation within this sample set at the five CpGs sites measured, including the known SNP sites rs141303113 and rs56105746, which overlap with CpG2 and CpG4, respectively. SNPs rs56377481, rs2020934 and rs2020935, which lie within the 300 bp *SLC6A4* DMR were also measured (Fig. 1b). Genotyping analysis excluded the presence of variation at rs56377481 (Table S5), but the influence of the higher frequency SNPs rs2020934 and rs2020935 on *SLC6A4* methylation was examined further (Table S6). There were differences in methylation at CpG1 (*p* = 0.0004) and CpG3 (*p* = 0.005) with respect to genotype at rs2020934, and in the methylation at CpG4 (*p* = 0.034) and CpG5 (*p* = 0.024) with respect to genotype at rs2020935. However, for both SNPs the median difference in methylation measurements at CpG sites was <2% (Table S7). The association between *SLC6A4* CpG5 methylation and total fat mass remained after adjusting the regression models for SNPs rs2020934 and rs2020935 (Table S8). Genotype alone was not significantly associated with any of the adiposity outcomes measured (Table S9).

### Maternal weight gain and parity is associated with altered infant *SLC6A4* methylation

To determine the potential maternal drivers of altered infant *SLC6A4* methylation, we examined the relationship between *SLC6A4* CpG5 methylation and maternal BMI, gestational weight gain, parity, smoking and Vitamin D status, five modifiable factors previously associated with increased infant adiposity [27–32]. Higher maternal pregnancy weight gain and lower parity were both associated with lower *SLC6A4* CpG5 methylation (*p* = 0.046; *p* = 0.029, respectively). There were no associations with pre-pregnancy maternal BMI, smoking or maternal vitamin D status (Table 3).

**Table 3:** Associations between maternal covariates and umbilical cord *SLC6A4*CpG5 methylation levels in the SWS cohort

Co-variate (continuous)	*n*	*r*_s_		*p*-value
Mother’s pre-preg BMI (kg/m^2^)	626	−0.028		0.48
Pregnancy weight gain (continuous)(kg/week)	593	−0.082		0.046*

Co-variate (categorical)	*n*	Group	Mean methylation (std error)	*p*-value
Low vitamin D (<50 nmol/L)	230	<50 nmol/L	78.63 (0.38)	0.602
	346	≥50 nmol/L	79.19 (0.28)	
Smoking during pregnancy	544	No	78.84 (0.24)	0.972
	85	Yes	79.00 (0.66)	
Parity	302	0	78.21 (0.36)	0.029*
	329	1+	79.47 (0.28)	
Pregnancy weight gain (categorical)	131	Inadequate	79.78 (4.82)	0.063
	186	Adequate	78.75 (5.95)	
	276	Excessive	78.36 (5.87)	

Association between continuous maternal factors; Mother’s pre-preg BMI and Pregnancy weight gain and SLC6A4 CpG5 methylation using Spearman correlations are shown. Associations between categorical maternal factors: Low vitamin D (<50 nmol/L), Smoking during pregnancy and parity using ranksum test are shown. Associations between categorical maternal factors: Pregnancy weight gain using Kruskal–Wallis test is shown

*EP* early pregnancy, *LP* late pregnancy

*p ≤ 0.01–0.05

Bold values indicate a *p* value lower than 0.05

### *SLC6A4* CpG5 methylation is associated with adiposity in adolescents at 17 years of age from the Raine Study

The methylation of *SLC6A4* CpG5 was measured in DNA from peripheral blood from adolescents aged 17 years from the RAINE cohort (*n* = 1200) (Table 4). Adjusting for age and sex, lower methylation of CpG5 was associated with greater BMI (*β* = −0.167 (−0.246, −0.088), *p* ≤ 0.001) waist circumference (*β* = −0.102 (−0.182, 0.0234), *p* = 0.011), subcutaneous fat (*β* = −0.136 (−0.216, −0.057), *p* = 0.001), subscapular skinfold thickness (*β* = −0.123 (−0.2015, −0.045), *p* = 0.002), abdominal skinfold thickness (*β* = −0.099 (−0.174, −0.025), *p* = 0.008), and suprailiac skinfold thickness (*β* = −0.1096 (−0.185, −0.034), *p* = 0.004) at age 17 years. Genotyping analysis showed no significant effects of rs2020934 and rs2020935 on CpG5 methylation in the RAINE cohort (Table S6). Blood cell counts were measured in these samples but strong collinearity (Table S10) between blood cell counts and BMI (variance inflation factors: 10.6–112.9) was observed, increasing the variance of the regression coefficients and making them unstable (Table S11). It was therefore not possible to adjust the association between methylation and BMI for cell type.

**Table 4:** Associations between peripheral blood SLC6A4 CpG5 methylation levels and measures of fat mass in children age 17 years from the RAINE cohort

Phenotype	Standardised CpG5
BMI at 17 years; standardised; adjusted for sex, age	
*n*	843
*β*	−0.167
*p*-value	<0.001**
95% CI	(−0.246, −0.088)
Waist circumference at 17 years; standardised; adjusted for sex, age	
*n*	813
*β*	−0.102
*p*-value	0.011*
95% CI	(−0.182, −0.0234)
Subcutaneous fat at 17 years; standardised; adjusted for sex, age	
*n*	774
*β*	−0.136
*p*-value	<0.001***
95% CI	(−0.216, −0.057)
Visceral fat; standardised; adjusted for sex, age	
*n*	648
*β*	0.025
*p*-value	0.557
95% CI	(−0.059, 0.111)
Subscapular skinfolds at 17 years; standarised; adjusted for sex, age	
*n*	785
*β*	−0.123
*p*-value	0.002**
95% CI	(−0.201, −0.045)
Abdominal skinfold at 17; standardised; adjusted for sex, age	
*n*	776
*β*	−0.099
*p*-value	0.008**
95% CI	(−0.174, −0.025)
Suprailiac skinfold at 17; standardised; adjusted for sex, age	
*n*	777
*β*	−0.1096
*p*-value	0.004**
95% CI	(−0.185, −0.034)

Associations between BMI, waist circumference, subcutaneous fat, visceral fat and skinfold thickness age 17 years are shown. 95% confidence limits (Cl) are shown

*Ln* natural logarithm

*p ≤ 0.01–0.05

**p ≤ 0.01, ***P ≤ 0.0001

Bold values indicate a *p* value lower than 0.05

### Lower *SLC6A4* methylation is associated with obesity in adipose tissue from adults in the BIOCLAIMS cohort

We next investigated whether SLC6A4 CpG5 was differentially methylated in subcutaneous adipose tissue of obese and lean adults from the BIOCLAIMS cohort (*n* = 65) (Table 1). Controlling for age and sex, obese individuals had lower *SLC6A4* CpG5 methylation (*p* = 0.019) and SLC6A4 mRNA expression (*p* = 0.008) in adipose tissue compared to lean individuals (Fig. 2d, Table S12). Genotyping analysis in all participants excluded the presence of SNPs at the cytosine of CpG5.

### In silco analysis of putative transcription factor binding sites across the DMR

CpG sites in close proximity are often co-regulated, however here, the CpG sites within the identified DMR of SLC6A4 show different and opposite directions of associations with measures of adiposity. We therefore carried out in silco analysis of the SLC6A4 DMR to identify the transcription factors that may bind across the CpGs of interest and potentially lead to their differential regulation. Matinspector analysis showed that the CpG sites of interest lay within the binding sites of a number of transcription factors, with CpG2 lying within a potential vitamin D-Retinoid X receptor (VDR-RXR) response element and CpG5 within a potential ZNF300 binding site (Table S13).

## Discussion

Our findings show that lower DNA methylation in umbilical cord and peripheral blood of a specific CpG site within the *SLC6A4* gene is associated with multiple measures of adiposity in infancy, childhood and adolescence. Moreover, in obese adults lower *SLC6A4* methylation in adipose tissue was accompanied by a change in SLC6A4 mRNA expression, suggesting that altered *SLC6A4* methylation may be of functional relevance in obesity.

The *SLC6A4* gene encodes the serotonin transporter, which has an important role in the regulation of emotion, behaviour, energy balance and appetite control. Alterations in *SLC6A4* expression have been associated with food intake and obesity in animals and humans; transgenic mice overexpressing *SLC6A4* are lighter and shorter than controls [33], whereas *SLC6A4* knockout mice develop late onset obesity, hepatic steatosis, glucose intolerance, and insulin resistance [34–36]. In humans, a promoter polymorphism within the *SLC6A4* gene results in the formation of either a long or a short allele [37]. The short allele reduces transcription compared to the long allele [38], and is associated with obesity in children [18], adult males [19] and with type-2 diabetes [38, 39]. Furthermore, in humans long-term use of SSRI (selective serotonin reuptake inhibitors), is associated with increased obesity and dyslipidaemia [40, 41].

Recent studies have suggested that serotonin has an important role in regulating metabolism in peripheral tissues. Serotonin receptors have been identified in virtually all organs. Adipocytes express a functional system for serotonin synthesis, reuptake and receptor activation, suggesting that serotonin may directly regulate adipocyte function [42]. Consistent with this, mice with a conditional adipose-specific knockout of tryptophan hydroxylase 1, the enzyme that catalyses the rate limiting step in serotonin biosynthesis, exhibit increased energy expenditure, reduced weight gain and decreased lipogenesis in white adipose tissue, suggesting that adipocyte-derived serotonin is important in energy homoeostasis [43].

In cord tissue from infants from the SWS cohort, methylation of *SLC6A4* CpG5 showed the strongest and most consistent association with measures of adiposity from birth through to 6 years of age. Two developmental pathways to obesity have been proposed; first, when foetal undernutrition is followed by rapid weight gain, and secondly, when foetal overnutrition is linked to greater adiposity at birth and beyond [22, 27]. Our findings suggest that lower *SLC6A4* CpG5 methylation, associated with higher adiposity from birth to 6–7 years, is a marker of the latter pathway. Three of these results passed a strict Bonferroni correction (*p* < 0.00083) in the SWS discovery cohort, notwithstanding that Bonferroni is likely overly conservative as the different measures of adiposity were highly correlated. Interestingly the associations between CpG5 methylation and total and % fat mass strengthened with age, suggesting an altered response to exposure to risk factors of obesity in later life, consistent with the paradigm of the developmental programming of obesity. Understanding how early life alters later life responses to an obesogenic environment, potentially through alterations in energy homoeostasis, appetite control or food choice will be critical for the development of effective intervention strategies. Weaker positive associations were also found between the methylation of *SLC6A4* CpGs 1 and 2 and % fat mass at 6–7 years. Developmentally induced changes in methylation are often CpG site specific [44], so this difference in the strength and direction of the association observed between the methylation of the CpG’s within the *SLC6A4* DMR may reflect different regulatory roles of the CpG sites within this region. SLC6A4 CpGs 1 and CpG5 are 135 bp apart and although CpG’s in close proximity are often co-regulated and methylation levels correlated, this is not what is observed for these sites. However, in silico analysis does show that these CpG sites are located within the response elements of different regulatory transcription factors, suggesting that they may be differentially regulated (Table S13).

In an independent cohort, lower methylation of *SLC6A4* CpG5 in peripheral blood was associated with multiple measures of adiposity at age 17 years, suggesting that *SLC6A4* methylation may be a robust marker of adiposity in peripheral tissue types across a range of ages from infancy to adolescence. Interestingly Zhou et al. [20] have reported that higher *SLC6A4* promoter methylation (−69 to –213 from the TSS) in peripheral blood leucocytes was associated with obesity in an adult monozygotic twin study. In our study using cord tissue, peripheral blood and adipose tissue, methylation of CpG5, located 1450 bp downstream of the TSS, was inversely associated with adiposity. This difference in the direction of the association between *SLC6A4* methylation and adiposity between the two studies may reflect the different location of the CpGs measured. Recent studies have shown that promoter and gene body methylation are inversely related, with high promoter methylation being associated with reduced transcription, and high gene body methylation positively associated with transcription [45]. Thus, the different direction of associations in the two studies may both reflect a lower level of *SLC6A4* transcription associated with increased adiposity. Consistent with this, we found lower *SLC6A4* CpG5 methylation in adipose tissue was accompanied by lower *SLC6A4* mRNA expression.

To date, the functional significance of altered *SLC6A4* methylation in peripheral blood cells or cord tissue is not known, although a number of studies have found that the methylation status of CpGs in peripheral tissues such as blood correlates with that of internal tissues [46, 47]. Interestingly, the methylation of specific CpGs in the promoter of *SLC6A4* in T cells and monocytes has been associated with in vivo measures of brain serotonin synthesis [48]. In agreement with this, we showed that differential methylation of *SLC6A4* in adipose tissue from a separate population was also associated with obesity. Although the downstream effects of reduced adipose tissue *SLC6A4* mRNA expression in our study are not known, this change would suggest an increase in peripheral serotonin availability in the obese state. Consistent with this, increased serum serotonin levels have been associated with obesity in mice [49].

The driving forces for the altered methylation of the *SLC6A4* DMR are currently unknown. Lower *SLC6A4* methylation was, however, associated with both higher maternal gestational weight gain and lower parity, maternal factors previously linked to increased offspring adiposity [28, 31]. Whether altered *SLC6A4* methylation lies on a causal pathway is not known. A substantial contribution to variation in methylation levels between individuals can also be a direct result of local genetic polymorphisms, so-called methylation quantitative trait loci (methQTL); the peak enrichment for distance across *cis* methQTLs has been experimentally determined as 45 bp [50]. In our study, the SNPs rs2020934 and rs2020935 were statistically associated with the methylation of several of the CpGs within the DMR of *SLC6A4*, but effect sizes were small and did not significantly affect the association between *SLC6A4* CpG methylation and measures of adiposity.

Our study has several limitations. First, we analysed DNA methylation in umbilical cord, blood and adipose tissue, which represent different cell populations with distinct epigenetic profiles. We measured blood cellular heterogeneity in the RAINE cohort, but here the cellular proportions strongly associated with measurements of adiposity. There was a particularly strong association between BMI and the peripheral blood neutrophil count, and previous studies have also reported a linear relationship between BMI and neutrophil number [51, 52]. Many studies have also shown increased neutrophil activation in obese subjects [53]. Multicolinearity was seen between all cell types and BMI for CpG5, overinflating the standard errors of the coefficients and making them unstable. Therefore adjusting the association between methylation and BMI for cellular proportions would violate the assumption of non-colinearity, which is a prerequisite for regression analysis. Consequently, we were unable to assess the dependence on cellular heterogeneity of the association of *SLC6A4* methylation in blood and BMI. We cannot rule out the possibility that the association between *SLC6A4* methylation in peripheral blood with adiposity in adolescence is dependent upon differences in cellular composition in blood. However, the existence of this association in different tissues, which are (in the case of adipose and cord) more homogeneous in their cellular composition, suggests that the relationship is not completely explained by cell type. In any case, our data showing that methylation status of *SLC6A4* CpG5 can distinguish levels of adiposity across a range of tissue types, suggests that altered methylation of this CpG maybe a valuable prognostic biomarker to identify individuals at risk and the efficacy of therapeutic interventions, especially as such biomarkers are likely to be measured in DNA from whole tissue samples rather than isolated specific cell types.

A second limitation of the study is that the number of participants in the SWS cohort with DEXA measurements at birth, 4 and 6 years and with sufficient DNA for methylation analysis did not completely overlap, so there are different numbers of participants at each time point. Moreover, there was also a limited availability of tissues in each cohort and in the SWS cohort, there were no measures of maternal glycemia, which has been suggested to be a driver of offspring epigenetic changes, so we could not determine whether infant’s SLC6A4 methylation was associated with maternal dysglycemia. Comparison of *SLC6A4* methylation across tissue types within the same individual would permit a greater understanding of tissue-specific differences in DNA methylation and the utility of SLC6A4 as a marker of future obesity. Thirdly, we did not have longitudinal methylation data, so we cannot ascertain whether methylation of *SLC6A4* changes during the life course or identify the factors that modify its methylation. However, this is the first study to define biomarkers of fat mass in perinatal tissue and replicate this result in the adipose tissue of adults from a separate cohort with associated alterations in gene expression. Therefore, not only is this epigenetic mark a potential biomarker of trajectory towards obesity, but it is also possible that its altered methylation may have functional consequences on energy balance, making it a potential target for intervention strategies to optimise health over the life course, or possibly to reverse obesity.

## Supplementary information

Tables S1-13
