# Supplementary material for: Differential SLC6A4 methylation: a predictive epigenetic marker of adiposity from birth to adulthood
Source: Int J Obes (Lond). 2019 Jan 8;43(5):974–88. doi: 10.1038/s41366-018-0254-3 (PMC6522375; doi:10.1038/s41366-018-0254-3)
Supplement: Supplementary file 1 — Tables S1-13 [file 41366_2018_254_MOESM1_ESM.docx]

**Additional Tables**

| **Primer** | **Sequence 5'→3'** | **Amplicon Length (bp)** | **Number of CpGs** |
| --- | --- | --- | --- |
| **Methylation Analysis:**  SLC6A4 CpG 1-2 Forward  SLC6A4 CpG 1-2 Reverse  SLC6A4 CpG 1-2 Sequencing  SLC6A4 CpG 3-5 Forward  SLC6A4 CpG 3-5 Reverse  SLC6A4 CpG 3-5 Sequencing  SLC6A4 CpG 5 Sequencing  **Genotyping Analysis:**  SLC6A4 CpG 1-2 CG SNP Forward  SLC6A4 CpG 1-2 CG SNP Reverse  SLC6A4 CpG 1-2 CG SNP Seq  SLC6A4 CpG 3-5 CG SNP Forward  SLC6A4 CpG 3-5 CG SNP Reverse  SLC6A4 CpG 3-5 CG SNP Seq  SLC6A4 CpG 5 G SNP Seq  SLC6A4 CpG 5 C SNP  SLC6A4 DMR SNP 2 Seq  SLC6A4 DMR SNP 4-5 Seq | AGGAAAAATGTTTAAGAAGGTGATATTG  Biotin-ACCTACTTCTCACTCATCCATATTA  GGATTTGTGTTTAATGAAATAG  GTTTTTAGGAGAGTAGGGAGTATATAGTTT  Biotin-ACCTACTTCTCACTCATCCATATTA  GTGTGAAGTTATTGAGG  GTATTTTGGGGTGGT  AGTGAACCTGGTCAATGGATTATT  Biotin- GAAGCAAGTAATGGGACAAAGAGT  TGAAACAGAGAACTGTGG  TTTGACTCTTTGTCCCATTACTTG  Biotin- AAACCTCATAAGAACCTGCTTCTC  TGTGTGAAGCCACTGAG  GCACTCTGGGGTGGC  CTCTGGGGTGGCAGT  AAGCCACTGAGGCCC  CAGTGACCGTTCCAA | 227  151  230  136 | 2  3  2  3 |

**Table S1. Primer sequences for methylation and genotyping Pyrosequencing assays.** PCR and sequencing primer sequences are shown for methylation and genotyping Pyrosequencing assays. 5’ Biotin labelled primers are indicated. Amplicon length and number of CpG’s within PCR amplicons are shown.

| **SWS SLC6A4 CpG** | **Obs** | **Mean** | **Std. Dev.** | **Min** | **Max** |
| --- | --- | --- | --- | --- | --- |
| **SLC6A4 CpG1**  **SLC6A4 CpG2**  **SLC6A4 CpG3**  **SLC6A4 CpG4**  **SLC6A4 CpG5** | 647  632  640  636  631 | 83.26456  84.44962  81.65252  85.83112  78.8664 | 3.619603  2.831814  4.42763  5.138124  5.639013 | 66.48  69.28  61.4  56.17  47.55 | 94.14  95.58  94.76  95.11  91.56 |

**Table S2. Summary Statistics for SLC6A4 CpG’s in the SWS Cohort**

| **Phenotype** |  | **CpG2**  **(Standardised)** | **CpG5**  **(Standardised)** | **Additional Adjustment** | **CpG2**  **(Standardised)** | **CpG5**  **(Standardised)** |
| --- | --- | --- | --- | --- | --- | --- |
| DXA: total fat at birth (standardised): adjusted for sex, age and gestational age | n  β  P-value  95% CI | 279  -0.0254  0.712  (-0.161, 0.110) | 279  -0.1261  **0.0463***  (-0.250, -0.002) | SLC6A4 batch effect, smoking during pregnancy, parity, IOM pregnancy weight gain and mother's pre-pregnancy BMI | 266  0.0099  0.884  (-0.124, 0.144) | 266  -0.0821  0.1867  ( -0.204, 0.040) |
| DXA: % fat at birth (standardised): adjusted for sex, age and gestational age | n  β  P-value  95% CI | 279  -0.0289  0.672  (-0.163, 0.105) | 279  -0.0962  0.1248  (-0.219, 0.027) | SLC6A4 batch effect, smoking during pregnancy, parity, IOM pregnancy weight gain and mother's pre-pregnancy BMI | 266  0.0037  0.956  (-0.130, 0.137) | 266  -0.0445  0.4726  (-0.166, 0.077) |
| DXA: total fat at 4 years (standardised): adjusted for sex | n  β  P-value  95% CI | 373  0.0288  0.564  (-0.069, 0.127) | 373  -0.1104  **0.0386***  (-0.215, -0.006) | SLC6A4 batch effect, smoking during pregnancy, parity, IOM pregnancy weight gain and mother's pre-pregnancy BMI | 346  0.0422  0.422  (-0.061, 0.145) | 346  -0.1301  **0.0196***  (-0.239, -0.021) |
| DXA: % fat at 4 years (standardised): adjusted for sex | n  β  P-value  95% CI | 373  0.0239  0.634  (-0.075, 0.122) | 373  -0.0853  0.1098  (-0.190, 0.019) | SLC6A4 batch effect, smoking during pregnancy, parity, IOM pregnancy weight gain and mother's pre-pregnancy BMI | 346  0.0255  0.629  (-0.078, 0.129) | 346  -0.0923  0.0992  (-0.202, 0.018) |
| DXA: total fat at 6 years (standardised): adjusted for sex and age | n  β  P-value  95% CI | 405  0.0658  0.168  (-0.028, 0.159) | 405  -0.2039  **0.0003****  (-0.314, -0.094) | SLC6A4 batch effect, smoking during pregnancy, parity, IOM pregnancy weight gain and mother's pre-pregnancy BMI | 383  0.0444  0.363  (-0.051, 0.140) | 383  -0.1611  **0.0058****  (-0.275, -0.047) |
| DXA: % fat at 6 years (standardised): adjusted for sex and age | n  β  P-value  95% CI | 404  0.0807  0.095  (-0.014, 0.176) | 404  -0.1447  **0.0113***  (-0.257, -0.033) | SLC6A4 batch effect, smoking during pregnancy, parity, IOM pregnancy weight gain and mother's pre-pregnancy BMI | 382  0.0543  0.272  (-0.043, 0.151) | 382  -0.0986  0.0946  (-0.214, 0.017) |

**Table S3. Multivariate analysis.** 1. Multivariate model of DXA (total & percentage) fat at birth, 4 and 6 years of age with *SLC6A4* CpG2 and CpG5 in the model, adjusted for *SLC6A4* batch effect. 2. Adjusted for SLC6A4 batch effect, smoking during pregnancy, parity, IOM pregnancy weight gain and mother's pre-pregnancy BMI (at initial visit). * p 0.01-0.05, ** p ≤ 0.01

| **Standardised triceps skinfold thickness adjusted for sex, age and gestational age** |  | **CpG1**  **(Standardised)** | **CpG2**  **(Standardised)** | **CpG3**  **(Standardised)** | **CpG4**  **(Standardised)** | **CpG5**  **(Standardised)** |
| --- | --- | --- | --- | --- | --- | --- |
| at birth (mm) | n  β  P-value  95% CI | 644  -0.00529  0.9091  (-0.096, 0.086) | 629  -0.04449  0.2762  (-0.125, 0.036) | 637  -0.0115  0.7701  (-0.089, 0.066) | 633  -0.1367  **0.0228***  (-0.254, -0.019) | 628  -0.09925  **0.0125***  (-0.177, -0.021) |
| at 6 months (mm) | n  β  P-value  95% CI | 602  -0.01269  0.7932  (-0.108, 0.082) | 587  0.01556  0.7129  (-0.067, 0.099) | 592  0.06781  0.109  (-0.015, 0.151) | 588  0.01138  0.8584  (-0.114, 0.137) | 584  -0.08961  **0.0382***  (-0.174, -0.005) |
| at 12 months (mm) | n  β  P-value  95% CI | 589  -0.01285  0.7946  (-0.110, 0.084) | 574  -0.01317  0.7596  (-0.098, 0.071) | 581  -0.00885  0.8313  (-0.090, 0.073) | 577  0.02306  0.7108  (-0.099, 0.145) | 572  -0.07812  0.0622  (-0.160, 0.004) |
| at 2 years (mm) | n  β  P-value  95% CI | 572  -0.00113  0.9818  (-0.098, 0.096) | 560  0.06316  0.1319  (-0.019, 0.145) | 566  -0.01203  0.7724  (-0.094, 0.070) | 563  -0.07535  0.2502  (-0.204, 0.053) | 557  -0.15507  **0.0003****  (-0.238, -0.072) |
| at 3 years (mm) | n  β  P-value  95% CI | 583  -0.02308  0.62659  (-0.116, 0.070) | 568  0.04458  0.29511  (-0.039, 0.128) | 575  0.00729  0.85907  (-0.073, 0.088) | 571  -0.05546  0.35796  (-0.174, 0.063) | 566  -0.16789  **0.00004****  (-0.248, -0.088) |
| at 6 years (mm) | n  β  P-value  95% CI | 550  -0.02184  0.6473  (-0.116, 0.072) | 537  0.0438  0.2988  (-0.039, 0.127) | 534  0.0226  0.5977  (-0.061, 0.107) | 530  0.01293  0.8393  (-0.112, 0.138) | 527  -0.10414  **0.0131***  (-0.186, -0.022) |

**Table S4. Associations between umbilical cord CpG SLC6A4 methylation and child’s skinfold thickness in the SWS cohort.** Associations between triceps skinfold thickness at birth, 6 months, 12 months, 2, 3 & 6 years, adjusted for sex, age, gestational age and SLC6A4 batch effect. 95% confidence limits are shown. * p 0.01-0.05, ** p ≤ 0.01

| **SNP ID** | **Genomic Location (GRCh37/hg19)** | **Alleles**  **(Reverse strand)** | **Minor Allele (R Strand) (Frequency in general population)** | **Minor Allele (Frequency in SWS population)** | **Minor Allele (Frequency in BIOCLAIMS population)** | **Minor Allele (Frequency in RAINE population)** |
| --- | --- | --- | --- | --- | --- | --- |
| rs141303113  rs56377481  rs56105746  rs2020934  rs2020935 | 28561577  28561509  28561490  28561460  28561455 | C/T (G/A)  C/G (G/C)  G/A (C/T)  A/G (T/C)  T/A (A/T) | T (A) (<0.01)  G (C) (<0.01)  A (T) (<0.01)  G (C) (0.5)  A (T) (0.08) | T (A) (0.00)  G (C) (0.00)  A (T) (0.00)  G (C) (0.51)  A (T) (0.07) | T (A) (0.00)  G (C) (0.00)  A (T) (0.00)  G (C) (0.43)  A (T) (0.02) | T (A) (0.00)  G(C) (0.00)  A(T) (0.00)  G (C) (0.45)  A (T) (0.05) |

**Table S5. Genomic variation located within the *SLC6A4* DMR and its frequency within the general, SWS and BIOCLAIMS populations.** The ID of the SNPs located within the *SLC6A4* DMR is shown alongside the alleles present and the frequencies of the minor allele within the general population, the SWS population and the BIOCLAIMS population. The reverse strand was used for all pyrosequencing, therefore the reverse strand alleles are shown in brackets.

| **Cohort** | **CpG** | **SNP** | **Genotype** | **Obs** | **P-value** | **SNP** | **Genotype** | **Obs** | **P-value** |
| --- | --- | --- | --- | --- | --- | --- | --- | --- | --- |
| **SWS** | 1 | rs2020934 | C/C  C/T  T/T | 148  305  143 | **0.0004**** | rs2020935 | A/A  A/T or T/T | 519  85 | 0.083 |
| **SWS** | 2 | rs2020934 | C/C  C/T  T/T | 147  296  140 | 0.064 | rs2020935 | A/A  A/T or T/T | 506  85 | 0.43 |
| **SWS** | 3 | rs2020934 | C/C  C/T  T/T | 152  296  141 | **0.005**** | rs2020935 | A/A  A/T or T/T | 512  85 | 0.304 |
| **SWS** | 4 | rs2020934 | C/C  C/T  T/T | 152  293  140 | 0.324 | rs2020935 | A/A  A/T or T/T | 509  84 | **0.034*** |
| **SWS** | 5 | rs2020934 | C/C  C/T  T/T | 149  290  141 | 0.474 | rs2020935 | A/A  A/T or T/T | 505  83 | **0.024*** |
| **RAINE** | 5 | rs2020934 | C/C  C/T  T/T | 406  423  98 | 0.32 | rs2020935 | A/A  A/T or T/T | 881  46 | 0.493 |

**Table S6. Effect of rs2020934 and rs2020935 genotypes on methylation of *SLC6A4* CpGs1-5 in the SWS Cohort and CpG5 in the RAINE cohort.** Kruskal-Wallis test on rs2020934 and methylation of *SLC6A4* CpGs 1-5, and Mann-Whitney ranksum test on rs2020935 and methylation of *SLC6A4* CpG 1-5 are shown for both the SWS and RAINE cohorts (CpG5 only for RAINE cohort). * p 0.01-0.05, ** p ≤ 0.01

| **SNP** | **CpG** | **Genotype** | **N** | **Median** | **IQR** |
| --- | --- | --- | --- | --- | --- |
| rs2020934 | 1 | C/C  C/T  T/T  Total | 148  305  143  596 | 84.605  83.74  82.75 | (81.81, 86.645)  (81.09, 85.7)  (80.24, 85.05) |
| rs2020934 | 3 | C/C  C/T  T/T  Total | 152  296  141  589 | 80.665  82.03  81.95 | (77.93, 83.235)  (78.805, 84.7)  (79.55, 84.5) |
| rs2020935 | 4 | A/A  A/T or T/T  Total | 509  84  593 | 87.7  86.585 | (82.12, 90.02)  (81.545, 89.35) |
| rs2020935 | 5 | A/A  A/T or T/T  Total | 505  83  588 | 79.74  78.41 | (76.77, 82.57)  (75.33, 81.11) |

**Table S7. *SLC6A4* CpG Methylation levels according to rs2020934 and rs2020935 Genotype in the SWS cohort.** Median methylation (%) and interquartile ranges are shown for methylation of *SLC6A4* CpGs 1-5 according to genotype. Only data from CpGs with differences by genotype at the 0.05 significance level are shown.

| **Regression term** | **DXA: total fat at birth (standardised) - adjusted for sex, age and gestational age** | | | **DXA: total fat at 4 years (standardised) - adjusted for sex** | | | **DXA: total fat at 6 years (standardised) - adjusted for sex and age** | | |
| --- | --- | --- | --- | --- | --- | --- | --- | --- | --- |
|  | n | **β** | **P-value** | n | **β** | **P-value** | n | **β** | **P-value** |
| CpG1  rs2020934 | 274 | 0.0665  0.0797 | 0.353  0.367 | 384 | -0.043  0.028 | 0.474  0.693 | 404 | 0.0548  0.0775 | 0.329  0.270 |
| CpG2  rs2020934 | 268 | -0.0084  0.0603 | 0.902  0.490 | 372 | 0.0354  0.0457 | 0.483  0.520 | 397 | 0.0795  0.0759 | 0.106  0.277 |
| CpG3  rs2020934 | 273 | -0.1238  0.1147 | **0.050***  0.193 | 382 | 0.002  -0.0161 | 0.969  0.822 | 401 | -0.0602  0.0667 | 0.254  0.349 |
| CpG4  rs2020934 | 270 | -0.1404  0.088 | 0.119  0.311 | 378 | -0.0093  -0.0128 | 0.905  0.857 | 399 | -0.1046  0.0556 | 0.194  0.428 |
| CpG5  rs2020934 | 270 | -0.1303  0.0873 | **0.026***  0.313 | 374 | -0.1227  -0.0084 | **0.018***  0.906 | 397 | -0.2162  0.078 | **0.0001****  0.264 |
| CpG1  rs2020935 | 279 | 0.0391  0.1641 | 0.586  0.358 | 385 | -0.0445  -0.0316 | 0.457  0.825 | 410 | 0.0377  0.0158 | 0.494  0.916 |
| CpG2  rs2020935 | 273 | -0.0296  0.1825 | 0.665  0.300 | 373 | 0.0348  -0.0674 | 0.491  0.636 | 403 | 0.0753  0.0275 | 0.123  0.855 |
| CpG3  rs2020935 | 278 | -0.0981  0.0465 | 0.117  0.799 | 383 | -0.0009  0.002 | 0.986  0.989 | 407 | -0.0517  0.0064 | 0.319  0.966 |
| CpG4  rs2020935 | 275 | -0.1217  0.0393 | 0.180  0.829 | 379 | -0.0171  -0.0297 | 0.827  0.840 | 405 | -0.0778  0.0013 | 0.317  0.993 |
| CpG5  rs2020935 | 275 | -0.1156  0.0417 | **0.049***  0.817 | 375 | -0.1314  -0.0519 | **0.011***  0.726 | 403 | -0.2178  -0.02 | **0.00009****  0.894 |

**Table S8. Associations between umbilical cord *SLC6A4* methylation and child’s total fat mass additionally adjusted for *SLC6A4* rs2020934 or rs2020935 in the SWS cohort.** Associations between *SLC6A4* CpG1-5 methylation and total fat mass at birth, 4 & 6 years is shown adjusted for SWS800 batch effect and *SLC6A4* rs2020934 or rs2020935. Findings were similar with multivariate SNP adjustment. rs2020934 coded as C/C=0, C/T=1, and T/T=2; rs2020935 coded as A/A=0 and A/T or T/T=1. * p 0.01-0.05, ** p ≤ 0.01

| **Phenotype** | **Coefficients** | **rs2020934** | **rs2020935** |
| --- | --- | --- | --- |
| DXA: total fat at birth (standardised): adjusted for sex, age and gestational age | n  β  P-value  95% CI | 289  0.0395  0.628  (-0.121, 0.200) | 294  0.1417  0.399  (-0.189, 0.472) |
| DXA: total fat at 4 years (standardised): adjusted for sex | n  β  P-value  95% CI | 407  -0.0139  0.839  (-0.149, 0.121) | 408  -0.0083  0.953  (-0.287, 0.271) |
| DXA: total fat at 6 years (standardised): adjusted for sex and age | n  β  P-value  95% CI | 422  0.0509  0.461  (-0.085, 0.187) | 428  0.0013  0.993  (-0.300, 0.303) |

**Table S9. Associations between *SLC6A4* rs2020934 and rs2020935 genotype and child’s total fat mass in the SWS cohort.** Associations between SLC6A4 rs2020934 or rs2020935 genotype and child’s total fat mass in the SWS cohort. Associations between genotype and total fat mass at birth, 4 & 6 years are shown. Fat outcomes have been standardised N (0,1). rs2020934 coded as C/C=0, C/T=1, and T/T=2; rs2020935 coded as A/A=0 and A/T or T/T=1.

| **Cell Sub type** | **Association with BMI** | |
| --- | --- | --- |
|  | **Pearson Correlation** | **Sig. (2-tailed)** |
| CD8T | -0.098 | 0.003 |
| CD4T | -0.082 | 0.015 |
| NK Cell | -0.079 | 0.018 |
| B Cell | -0.079 | 0.018 |
| Monocyte | 0.043 | 0.201 |
| Granulocyte | 0.160 | 0.001 |

**Table S10 Association between cellular type and BMI in the RAINE cohort.** N = 894

| **Phenotype** | **CD8T** | **CD4T** | **NK Cell** | **B Cell** | **Mono** | **Gran** |
| --- | --- | --- | --- | --- | --- | --- |
| BMI at 17 years Ln transformed; adjusted for sex, age | 23.8 | 56.7 | 26.8 | 13.4 | 11.2 | 109.6 |
| Waist Circumference at 17 years Ln transformed; adjusted for sex, age | 24.5 | 57.2 | 27.9 | 13.3 | 11.1 | 110.1 |
| Subcutaneous Fat at 17 years Ln transformed; adjusted for sex, age | 24.3 | 57.3 | 28.0 | 13.6 | 11.4 | 109.8 |
| Visceral Fat Ln transformed adjusted for sex, age | 24.8 | 57.6 | 29.5 | 14.0 | 11.4 | 112.9 |
| Subscapular skinfolds at 17 Ln Transformed; adjusted for sex and age | 24.4 | 55.4 | 13.2 | 10.7 | 28.3 | 106.2 |
| Abdominal skin fold at 17 adjusted for sex and  age | 24.1 | 55.4 | 28.1 | 13.1 | 10.6 | 106.3 |
| Suprailiac skin fold at 17 adjusted for sex and  age | 24.2 | 55.5 | 28.2 | 13.1 | 10.6 | 106.4 |

**Table S11. Variance Inflation Factors for *SLC6A4* CpG5 methylation values and cellular type in the RAINE cohort**

| **Variable** |  | **Obesity according to BMI** |
| --- | --- | --- |
| SLC6A4 Expression | P-value  OR  95% Cl | **0.008****  0.040  (0.004, 0.434) |
| SLC6A4 CpG5 Methylation | P-value  OR  95% Cl | **0.019***  0.903  (0.829, 0.983) |

**Table S12. Associations between *SLC6A4* gene expression or *SLC6A4* CpG5 methylation and Obesity in the BIOCLAIMS cohort according to BMI.** Participants are grouped into lean and obese according to BMI. Odds ratio (OR) and 95% confidence limits are shown from binary logistic regressions. * p 0.01-0.05, ** p ≤ 0.01.

|  | **SLC6A4 CpG 1** | **SLC6A4 CpG 2** | **SLC6A4 CpG 3** | **SLC6A4 CpG 4** | **SLC6A4 CpG 5** |
| --- | --- | --- | --- | --- | --- |
| **Response Elements Present Over CpG** | NRSF 02  ATF 01  WHN 01 | VDR_RXR 03  CDX 01  HOXC13 02  PRDM5 01 | NUDR 01  CREL 01  NF1 03 | E2F 01  NGFIC 01  AHR 01  ATF6 01  HAS 01  STAF 01  CTCF 01  ROAZ 01 | ZNF300 01  ER 03  MYBL1 01  VMYB 05  BRN2 01  MEIS1A_HOX9  YY2 01  CDP 02  PAX1 01  CLOX 01 |

**Table S13. Transcription Factors predicted to bind to CpG’s 1-5 within the *SLC6A4* DMR.** Only response elements which cover the CpG of interest are shown.
